# Metabolic analyses reveal dysregulated NAD^+^ metabolism and altered mitochondrial state in ulcerative colitis

**DOI:** 10.1371/journal.pone.0273080

**Published:** 2022-08-17

**Authors:** Yu Hui Kang, Sarah A. Tucker, Silvia F. Quevedo, Aslihan Inal, Joshua R. Korzenik, Marcia C. Haigis

**Affiliations:** 1 Department of Cell Biology, Blavatnik Institute, Harvard Medical School, Boston, MA, United States of America; 2 Division of Gastroenterology, Hepatology and Nutrition, Boston Children’s Hospital, Boston, MA, United States of America; 3 Division of Gastroenterology, Hepatology and Endoscopy, Brigham and Women’s Hospital, Boston, MA, United States of America; 4 Department of Medicine, Harvard Medical School, Boston, MA, United States of America; Duke University, UNITED STATES

## Abstract

Ulcerative colitis (UC) is a complex, multifactorial disease driven by a dysregulated immune response against host commensal microbes. Despite rapid advances in our understanding of host genomics and transcriptomics, the metabolic changes in UC remain poorly understood. We thus sought to investigate distinguishing metabolic features of the UC colon (14 controls and 19 patients). Metabolomics analyses revealed inflammation state as the primary driver of metabolic variation rather than diagnosis, with multiple metabolites differentially regulated between inflamed and uninflamed tissues. Specifically, inflamed tissues were characterized by reduced levels of nicotinamide adenine dinucleotide (NAD^+^) and enhanced levels of nicotinamide (NAM) and adenosine diphosphate ribose (ADPr). The NAD^+^/NAM ratio, which was reduced in inflamed patients, served as an effective classifier for inflammation in UC. Mitochondria were also structurally altered in UC, with UC patient colonocytes displaying reduced mitochondrial density and number. Together, these findings suggest a link between mitochondrial dysfunction, inflammation, and NAD^+^ metabolism in UC.

## Introduction

The role of mitochondrial function in Inflammatory Bowel Disease (IBD) has become an active area of investigation in recent years, particularly in ulcerative colitis (UC). Several studies have established that mitochondrial dysfunction is a hallmark of IBD pathology [1–3]. The intestinal mucosa in IBD is characterized by hypoxia and increased oxidative stress, and a variety of genes involved in mitochondrial function such as *CUL2*, *LACC1* and NADPH oxidase have been implicated in IBD [4–8]. Suppression of mitochondrial gene expression and function have also been observed in a large UC cohort [2]. However, the mechanism by which dysfunctional mitochondrial metabolism influences IBD pathogenesis remains unclear.

Traditionally perceived as cellular hubs of energy production in the form of adenosine triphosphate (ATP), mitochondria are now recognized as dynamic organelles with diverse roles in regulating metabolism and various cell functions, including apoptosis, production of reactive oxygen species, regulation of gene expression, and modulation of signaling pathways [9–11]. Mitochondrial dysfunction has thus been implicated in multiple metabolic and age-related diseases such as diabetes, cancer, and neurodegenerative disease [12].

In this study we examined changes in mitochondrial morphology in colon tissues from UC patients. Furthermore, we performed metabolomic analysis to identify underlying metabolic alterations in patients with UC that may be linked to mitochondrial dysfunction.

## Materials and methods

### Study design and sample collection

Colonic biopsies were obtained at colonoscopy with biopsy forceps from individuals with active UC, UC in remission and controls undergoing colonoscopy for colon cancer screening. Tissue biopsies were obtained from the sigmoid region and immediately placed in liquid nitrogen. Signed informed consent was obtained from participants prior to providing samples. Inflammation was determined by histologic assessment by a pathologist. Activity within the disease cohort was defined as inflammation in any part of the colon as determined by pathology. All research in this study involving human participants has been approved under the Mass General Brigham IRB (Protocol #2010P002317).

### Metabolomics–Sample preparation

Colonic biopsies (1–15 mg) were snap frozen and metabolites extracted by methanol-chloroform extraction. Briefly, tissues were resuspended in 600 μl of 60% methanol and 400 μl of chloroform. All solvents used during sample preparation were of LC-MS-grade quality. 2 stainless steel beads were added and the tissues homogenized using a TissueLyser LT (Qiagen) for 2 min at 50 Hz. The extracts were centrifuged at 13.3 krpm, 4°C for 15 min and the aqueous layer dried in a SpeedVac. The dried extracts were then reconstituted in 1:1 acetonitrile-water for analysis on the MS.

Samples were run on a Vanquish UHPLC system (Thermo Fisher) coupled to a Q-Exactive HF-X mass spectrometer utilizing a HESI probe (Thermo Fisher) on both positive and negative ion modes. For the LC, hydrophilic interaction liquid chromatography (HILIC) was used, specifically a 150 X 2.1 mm iHILIC®-(P) Classic polymeric column equipped with a 2.1 x 20 mm iHILIC®-(P) Classic Guard column (both 5 μm, 200 Å, HILICON AB). Buffers A (20 mM ammonium carbonate in water, with 0.1% ammonium hydroxide for negative mode or formic acid to pH 6.8 for positive mode) and B (100% acetonitrile) were used. A linear gradient was performed at a flow rate of 0.15 ml/min as follows: 80% to 20% Buffer B linear gradient from 0–20 min, 20% to 80% Buffer B linear gradient from 20–20.5 min, hold at 80% Buffer B from 20.5–28 min, hold at 80% Buffer B from 28–30 min without data acquisition. The column was held at 25°C, the column preheater at 30°C and the autosampler at 10°C. MS acquisition was performed on full scan mode over a range of 70–1000 m/z and a resolution of 60,000, an AGC target of 1e5 and a maximum injection time of 20 ms with a 5 eV in-source CID. The spray voltage was set to 3.9 kV (positive) or 3 kV (negative), the heated capillary was set at 275°C and the probe set at 325°C (positive) or 350°C (negative). The sheath gas flow rate was 40, the auxiliary gas was set at 10 (positive) or 15 (negative) and the sweep gas flow rate set to 1.

### Metabolomics–data analysis

Feature extraction and peak integration was performed using Tracefinder version 4.1 (Thermo Fisher). Metabolites were identified using exact mass with a 5 ppm tolerance and retention time with reference to an in-house library of chemical standards. Allowed species were [M+H]^+^ for positive mode and [M-H]^-^ for negative mode. Peaks were manually curated to exclude low quality peaks (e.g. noise, multiple peaks) and metabolites with no identified peaks. For metabolites detected in both modes, the peaks in negative mode were selected. Based on these criteria, 75 metabolites were consistently detected across both experiments and the peak areas used for further analysis on RStudio IDE and Graphpad Prism.

Data processing was performed for each experiment in each mode using code derived from Metaboanalyst [13]. For univariate analyses (single metabolite comparisons, volcano plot), the areas were normalized to a constant sum (1000).

For multivariate analyses (PCA, Pathway analysis), constant sum normalization was performed and metabolites with constant values removed. Missing or zero values were replaced with half of the minimum positive value. The values were then glog transformed ($\text{g}\;\log_{2}(x)=\log_{2}\frac{x+\sqrt{x^{2}+{\left(minimum\;value\right)}^{2}}}{2}$) and autoscaled. Data for both experiments were then combined and autoscaled to account for experiment-to-experiment variation and rescaled after outlier removal. Pathway analysis was performed using metPA based on a global test, with node importance assessed by relative betweenness centrality [14].

Statistical significance was assessed using the Mann-Whitney test with p value < 0.05 considered as significant.

The following R packages were used for these analyses: *tidyverse*, *rstatix*, *EnhancedVolcano*, *PCAtools*, *factoextra*, *RColorBrewer*, *Hmisc*, *corrplot*.

### Mitochondrial electron microscopy–sample preparation, data collection, and analysis

Colonic biopsies (cut into 1–2 mm cubes) provided by the Crohn’s and Colitis Center at Brigham and Women’s Hospital were fixed with 0.1 M cacodylate buffer, pH 7.4, containing 2.5% glutaraldehyde and 2% paraformaldehyde, and stored at 4°C for up to 2 weeks prior to further processing. Fixed samples were submitted to the Harvard Medical School Electron Microscopy Facility for embedding, sectioning, and staining. Imaging was done using a Tecnai G^2^ Spirit BioTWIN Transmission Electron Microscope equipped with an AMT 2k CCD camera. 30–40 electron micrographs were taken per patient sample at three different magnifications, 1200x, 4800x, and 9600x.

For a given biopsy, 1200x images were used to define areas where colonocytes were present, and 4800x and 9600x images were used to quantify mitochondrial features of interest. Fiji software was used to identify individual mitochondria and determine their features, including area and roundness (approximated by mitochondrial ratio of length to width). When calculating total area in biopsy tissue occupied by mitochondria, the area of any lumen included in the defined analysis area was subtracted to obtain the sample area to ensure our calculations reflected only biopsy tissue area. All analysis was performed blinded to which samples were from controls versus UC patients. Graphpad Prism was used for statistical analyses. Statistical significance was assessed using the Mann-Whitney test with p value < 0.05 considered as significant. Values of technical replicates were averaged for each sample prior to statistical analysis.

## Results

**Fig 1:**
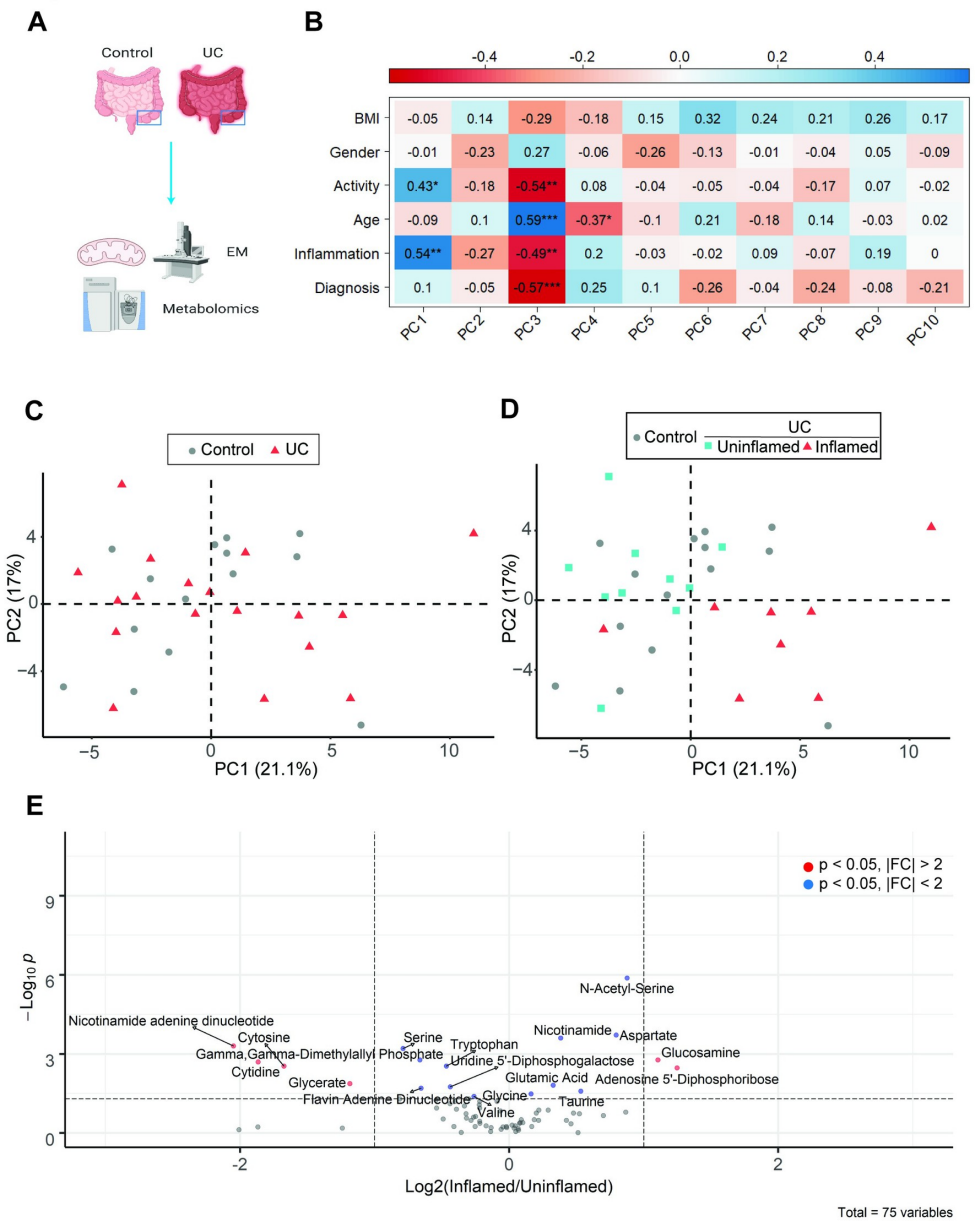
Metabolomic profiles of intestinal tissue cluster primarily by inflammation rather than diagnosis. **(A)** Schematic of experimental design. Created with Biorender. Colonic tissue biopsies were subjected to metabolomic analysis across 2 independent experiments or mitochondrial structure analysis by electron microscopy. **(B)** Pearson correlation analysis of PCs and various metadata, with * p<0.05, **p<0.01, ***p<0.001 **(C-D)** Principal component analysis (PC1 vs PC2) of metabolomic data comparing **(C)** Control vs UC biopsies or **(D)** control vs UC biopsies further stratified by inflammation state. **(E)** Volcano plot of metabolites differentially regulated in uninflamed (control + UC) vs inflamed UC tissues, plotting log_2_(inflamed/uninflamed) vs -log_10_(P) value as assessed by Mann-Whitney test.

To assess the metabolic differences that might drive mitochondrial dysfunction in UC, we collected biopsies from the sigmoid colon of controls (n = 14) and UC patients (n = 19) and subjected them to mitochondrial electron microscopy and metabolomics analysis **(Fig 1A)**. The characteristics of these patients are summarized **(Table 1 and S1A Fig)**. 11 of the 19 UC patients, 7 of whom were in remission (i.e. inactive), had biopsies taken from uninflamed regions of the colon **(Table 1**). 8 patients, all of whom had active UC, had samples collected from inflamed regions of the colon (**Table 1**). We note that the control group was significantly older than UC patients with inflamed tissue as these control biopsies were collected as part of routine colon screenings **(Table 1)**.

**Table 1:** Patient demographics showing their age (mean ± S.D.), gender, BMI (mean ± S.D.), biopsy location, duration of disease (mean ± S.D.), disease activity, and extent of disease (mean ± S.D.). Activity refers to the presence of inflammation in any region of the colon and is not restricted to the site of the tissue biopsy, while inflammation refers specifically to the site of the tissue biopsy. ***p<0.001 by Kruskal-Wallis test with Dunn’s correction for multiple comparisons.

	Controls (n = 14)	UC Uninflamed (n = 11)	UC Inflamed (n = 8)
Age	58.6 ± 6.0	47.5 ± 12.9	36.4 ± 5.8***
Gender (%M)	35.7%	72.7%	62.5%
BMI	25.81 ± 6.5	27.01 ± 4.8	27.03 ± 4.6
Biopsy location	Sigmoid	Sigmoid	Sigmoid
Duration of disease (years)	N/A	14 ± 9.2	12 ± 8.5
Active	0	4 (36.4%)	8 (100%)
*Extent of disease*			
Pancolitis	N/A	7 (63.6%)	5 (50%)
Ulcerative proctitis	N/A	1 (9.1%)	2 (25%)
Left-sided	N/A	3 (27.3%)	2 (25%)

### Intestinal metabolomic profiles cluster primarily by inflammation state

To gain insight into the metabolic state of human UC, biopsies of colon tissues from UC patients were subjected to metabolic profiling across 2 independent experiments. Using a semi-targeted approach, we consistently detected 75 metabolites, whose intensities were normalized to a constant sum as the sum of raw intensities correlated with the initial weight of the biopsy **(S1B Fig)**. Initial principal component analysis (PCA) attributed most variation to 1 sample, hence this outlier was removed in subsequent analyses (**S1C Fig**).

We first analyzed what clinical factors might drive the metabolic differences by performing a correlation analysis between the principal components (PC) and various clinical parameters **(Fig 1B)**. Focusing on PC1-2, which captured most (~38.1%) of the variation, we observed no statistically significant correlation between the PCs and the parameter “diagnosis”, which is defined as whether subjects were control or UC patients **(Figs 1B and S1D)**. This finding was supported by the degree of overlap between controls and UC patients on the PCA plot **(Fig 1C)**. Interestingly, the metabolite profiles correlated significantly with the parameter “Inflammation”, which is the inflammation state of the tissue **(Fig 1B)**. Indeed, inflamed colon tissues formed a distinct cluster from controls and patients with uninflamed tissues **(Fig 1D)**. Similar results were observed with regard to activity status **(Figs 1B and S1E)**.

Together, these data suggest that inflammation, rather than UC diagnosis, was the primary driver of metabolic differences.

### NAD^+^ metabolism is dysregulated in inflamed tissue

We next sought to understand the metabolites driving these differences. As the metabolic profiles of control and uninflamed patient tissues were similar **(Fig 1D)**, these groups were combined for differential analysis, revealing several significantly altered metabolites between inflamed and uninflamed tissue **(Figs 1E and S2A–S2O)**.

**Fig 2:**
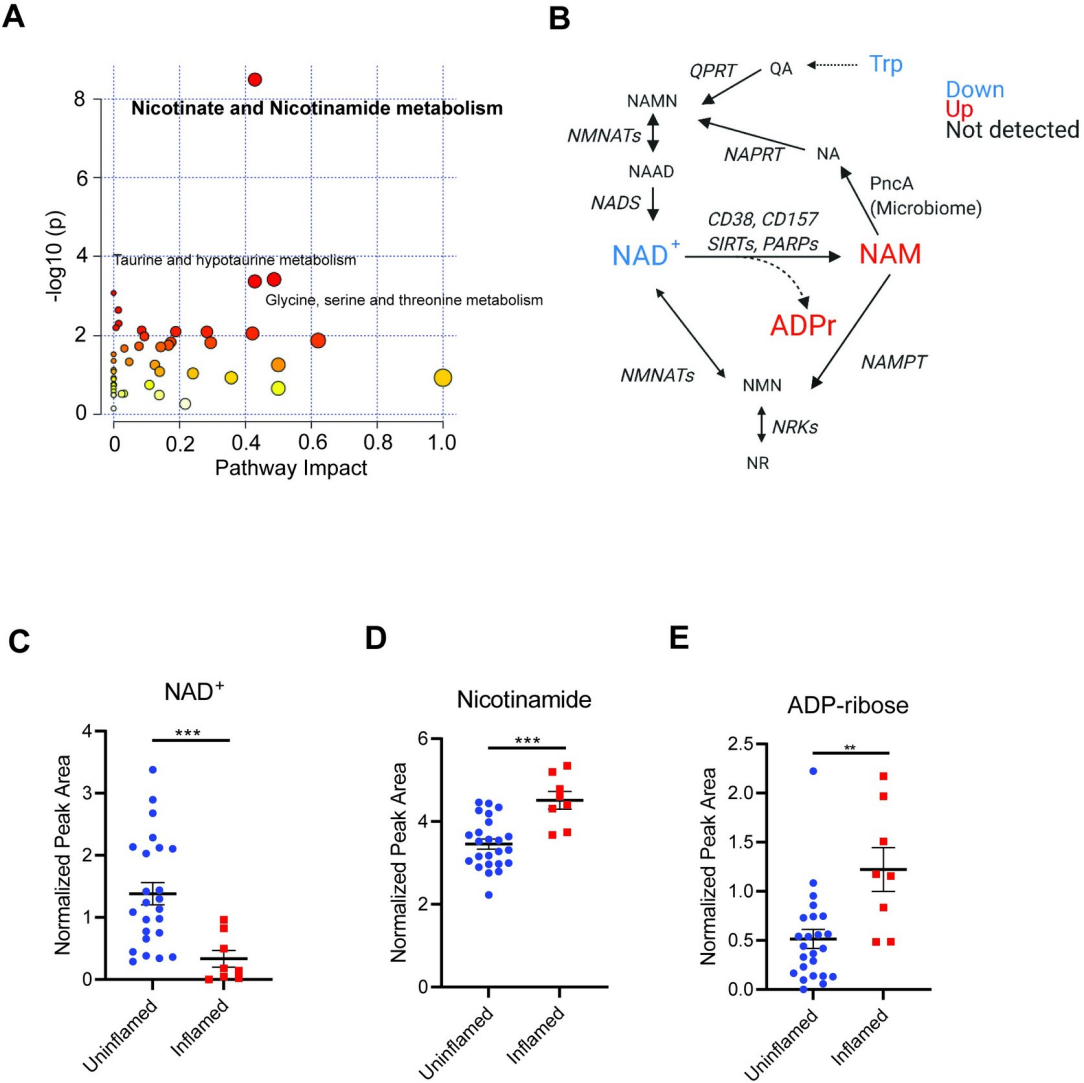
Pathway analysis identifies dysregulation of NAD^+^ metabolism in inflamed UC tissue. **(A)** Metabolic pathway (MetPA) analysis of pathways differentially regulated in uninflamed (control + UC) vs inflamed UC colon tissue biopsies, with top significant pathways indicated. **(B)** NAD^+^ metabolism pathways, with differentially regulated metabolites color-coded (p<0.05, Mann-Whitney test). **(C)–(E)** Normalized abundance of **(C)** NAD^+^ and its degradation products **(D)** NAM and **(E)** ADPr indicated, analyzed by Mann-Whitney test. Graphs depict mean ± SEM. **p<0.01, ***p<0.001. NAD^+^—Nicotinamide adenine dinucleotide, NAM—Nicotinamide, ADPr–ADP ribose, NA–Nicotinic Acid, NR—nicotinamide riboside, NAMN- nicotinic acid mononucleotide, NAAD—nicotinic acid adenine dinucleotide, NMN—nicotinamide mononucleotide, QA–Quinolinic acid, NMNAT- nicotinamide mononucleotide adenylyltransferase, NAPRT–nicotinate phosphoribosyltransferase, NADS—NAD synthetase, NRK–Nicotinamide riboside kinase, SIRT–Sirtuins, PARP–Poly(ADP-ribose) polymerase, NAMPT—Nicotinamide phosphoribosyltransferase, PncA–Nicotinamidase.

Pathway analysis revealed “nicotinate and nicotinamide metabolism” as the top regulated pathway distinguishing inflamed tissues from uninflamed tissues **(Fig 2A)**. NAD^+^ is a critical metabolite that not only harvests energy from catabolic processes but also serves as a cofactor for multiple metabolic enzymes [15]. NAD^+^ can be degraded by multiple enzymes (SIRTs, PARPs, CD38, CD157) to generate NAM and ADPr or its variants (2/3’-O-acyl-ADPr, poly(ADPr), cyclic-ADPr) depending on the enzyme [15]. NAD^+^ can be synthesized via various pathways—either from NAM, nicotinamide riboside (salvage pathway), tryptophan (*de novo* pathway) or from nicotinic acid (Preiss-Handler pathway) [16] **(Fig 2B)**.

Consistent with the pathway analysis, we observed that inflamed tissues displayed reduced levels of NAD^+^ **(Fig 2C)** and elevated levels of the corresponding metabolic by-products NAM **(Fig 2D)** and ADPr **(Fig 2E)**. Within uninflamed tissues, control and UC uninflamed tissues displayed no differences in these metabolites, while inflamed tissues displayed reduced NAD^+^ compared to control and UC uninflamed tissues and elevated NAM and ADPr compared to controls (**S3A–S3C Fig**). Together, these data are consistent with the idea that that NAD^+^ degradation or recycling is altered during intestinal inflammation.

Our PC correlation analysis also revealed associations between PC3/PC4 and several clinical parameters (**Fig 1B**), although these contributed less to overall metabolic variance (**S1D Fig**). In agreement with this analysis, we observed distinct clusters on PC3/4 according to diagnosis (**S4A Fig**), inflammation (**S4B Fig**), activity (**S4C Fig**) and age (**S4D Fig**). To distinguish which metabolites might be driving the separations in PCA, we analyzed the loadings (top/bottom 5% of variables) for each PC. Focusing on PC3-4, loadings analysis revealed several metabolites (including NAM), suggesting that these metabolites might be associated with the clinical parameters (**S4E Fig**). However, it is difficult to distinguish whether these metabolites are associated with one or several parameters. This is further complicated by the association of these parameters with one another as a result of disease biology (inflamed tissues are all from active UC patients) or experimental design (controls undergoing colonoscopy will typically be of older age) (**S4F Fig**). We analyzed the loadings for PC1, the component associated with inflammation and activity only, as well as PC2 (**Fig 1B**). This analysis revealed NAD^+^ to be negatively associated with PC1 (**S4G Fig**). Therefore, the depletion of NAD^+^ is likely to be a feature specific to disease pathogenesis.

### Intestinal NAD^+^/NAM ratios predict inflammation but not diagnosis

**Fig 3:**
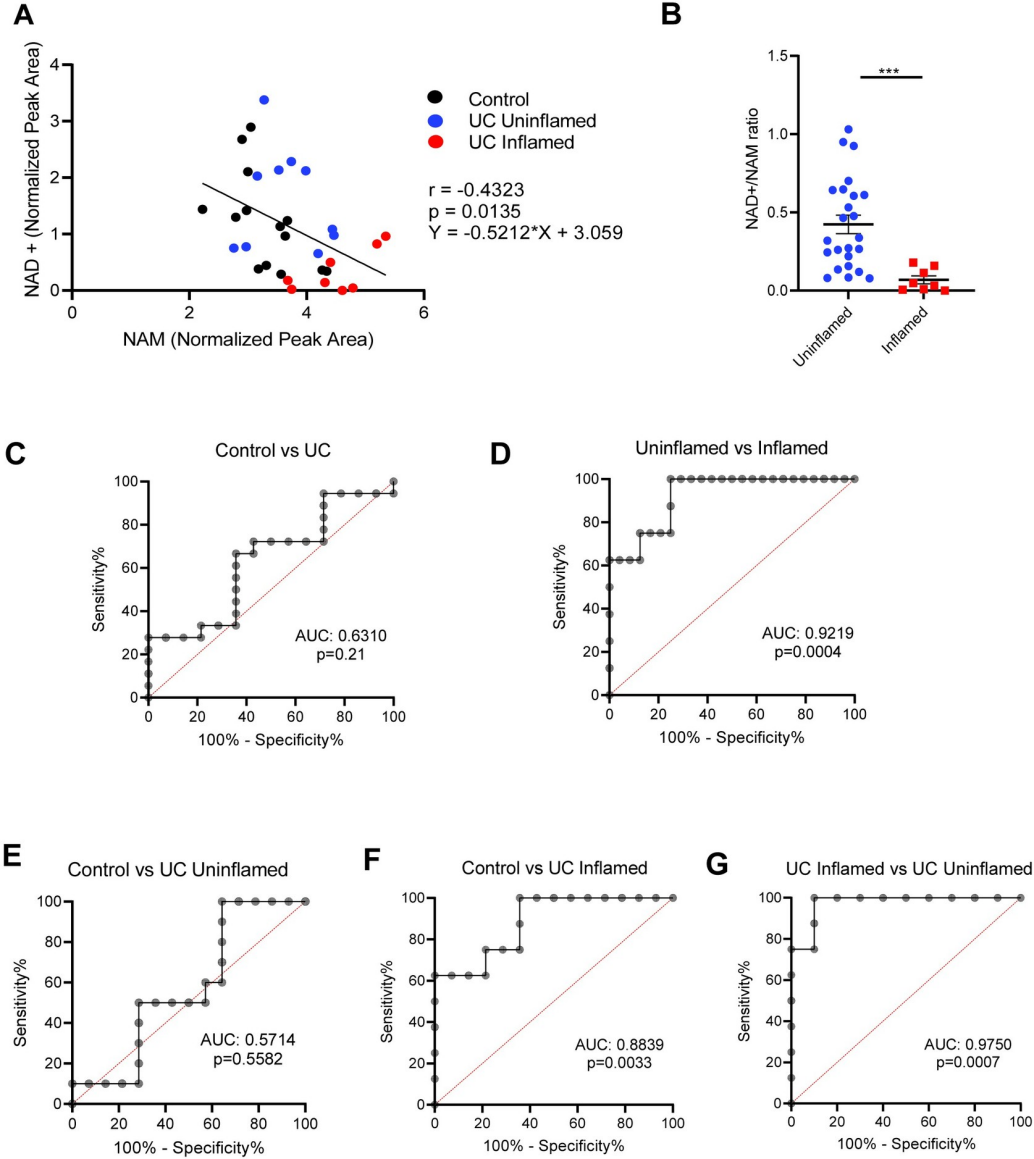
Intestinal NAD^+^/NAM ratios predict inflammation but not diagnosis. **(A)** Pearson correlation analysis of normalized NAD^+^ vs NAM levels in various disease states. **(B)** Comparison of NAD^+^/NAM ratios in inflamed vs uninflamed (control + UC) tissues. Graph depicts mean ± SEM, *** p<0.001 by Mann-Whitney test. **(C-G)** Receiver operating characteristic (ROC) analysis of the ability of colon biopsy NAD^+^/NAM ratio to predict various disease states, with area under the curve (AUC) and p values shown. Specifically, they show the ability of NAD^+^/NAM ratios to distinguish **(C)** diagnosis, **(D)** inflammation state, **(E)** control vs uninflamed UC tissue, **(F)** control vs inflamed UC tissue and **(G)** inflamed vs uninflamed UC tissue.

Consistent with NAM being a product of NAD^+^ degradation, we observed that NAD^+^ levels inversely correlated with NAM levels **(Fig 3A)**. We thus hypothesized that the NAD^+^/NAM ratio, which was reduced in inflamed tissues as expected **(Fig 3B)**, might predict inflammation state in the intestine.

Receiver operating characteristic (ROC) analysis revealed that NAD^+^/NAM ratio could discriminate based on colon tissue inflammation but could not distinguish controls from UC patients **(Fig 3C and 3D)**. We also observed that, within uninflamed tissues, the NAD^+^/NAM ratio could not discriminate between control and UC samples **(Fig 3E)**. However, inflamed tissues from UC patients could be discriminated from control or uninflamed UC samples **(Fig 3F and 3G)**. Together, these data suggest that the NAD^+^/NAM ratio can be used as a predictor of inflammation in UC.

### UC patients display altered mitochondrial morphology in the intestine

Defects in NAD^+^ metabolism have been implicated in mitochondrial dysfunction [16]. We hypothesized that mitochondrial dysfunction might be a distinguishing feature of IBD.

**Fig 4:**
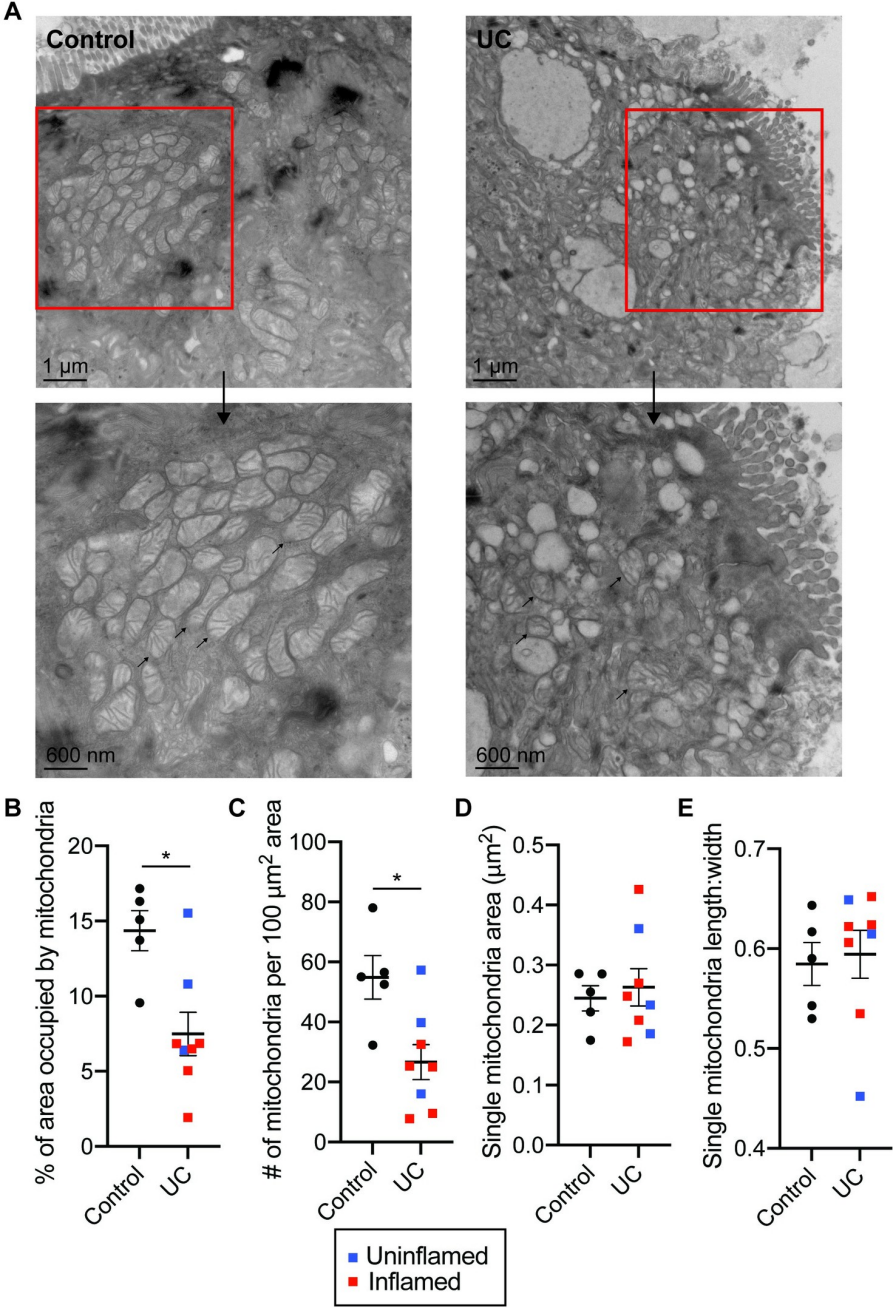
Mitochondrial abnormalities in UC patients. **(A)** Representative EM micrographs of colon biopsy tissue from controls and UC patients, with a scale bar of 1 μm. Rectangle indicates region represented in lower panels, with a scale bar of 600 nm. Arrows indicate individual mitochondria. **(B-E)** Quantitation of the EM micrographs (n = 4 images per sample, 5–8 samples per group) showing changes in **(B)** the percent area occupied by the mitochondria between groups and **(C)** the number of mitochondria per 100 μm^2^ area, with no significant changes in **(D)** single mitochondrial area or **(E)** the roundness of mitochondria. Graphs depict mean ± SEM, *p<0.05 by Mann-Whitney test. Each point denotes the average of technical replicates for one control/patient.

To test this idea, we assessed mitochondrial morphology in the colonic tissue of controls and UC patients by electron microscopy (EM). Most of these samples were from patients who contributed tissues for metabolomics analysis (metadata in **S1 Table**). We observed striking differences in mitochondrial number and morphology in colonocytes from UC patients (**Fig 4A**). Quantification analyses revealed a significant reduction in mitochondrial density and number (**Fig 4B and 4C**), indicative of mitochondrial dysfunction. These differences tended to be more pronounced in inflamed UC tissues compared to uninflamed UC tissues, but more samples will be required to validate these differences (**Fig 4B and 4C**). We did not observe significant differences in the size or roundness of individual mitochondria **(Fig 4D and 4E)**. Similar differences were also observed when comparing uninflamed (control + UC uninflamed) with inflamed samples (**S5 Fig**).Together, these suggest a potential role for mitochondrial dysfunction in IBD pathogenesis.

## Discussion

It is increasingly appreciated that metabolic alterations play a key role in shaping the pathogenesis of IBD and can serve as useful biomarkers [17, 18]. Although metabolomics have been performed in earlier IBD studies yielding useful insights, most of these studies were run on biofluids (stool, urine, blood) [19–23] and it is unclear whether these changes can be translated to the gut. We performed metabolomics in the UC colon and show that NAD^+^ depletion is a key feature of UC patients with active colonic inflammation. NAD^+^ depletion is a hallmark of aging and aging-related metabolic diseases such as fatty liver disease, diabetic nephropathy, and neurodegenerative disorders [15]. Our study adds IBD, specifically UC, to this list of diseases [24, 25]. Furthermore, the specificity of NAD^+^ depletion for inflamed tissues suggests that it might play an important role in regulating disease outcome.

We provide evidence that the NAD^+^ depletion likely stems from enhanced NAD^+^ degradation, as we also observed an increase in NAM and ADPr—key metabolic products of NAD^+^ catabolism—in inflamed UC. While elevations in NAM levels have been observed in active UC [26], the source of this increase was not explored. Of the enzymes involved in NAD^+^ catabolism (SIRTs, PARPs, CD38, CD157), CD38 is an attractive target for further investigation as it can directly generate NAM and ADPr from NAD^+^ [27, 28]. Upregulation of CD38 has been reported in human IBD, specifically in inflamed tissues [29]. Moreover, *Cd38*^*-/-*^ mice display milder colitis, suggesting that the upregulation of this molecule is pathogenic in IBD and that therapeutic modalities targeting CD38 in cancer and aging might also be relevant for this disease [30–32]. However, we do not exclude the possibility that ADPr might be derived from further breakdown of 2/3’-O-acyl-ADPr produced by SIRT activity and/or poly(ADPr) produced by PARP activity [33]. Further studies assessing the roles of these pathways in the UC colon will be important for defining the pathways involved in UC.

It has been reported that UC patients display reduced expression of multiple mitochondrial genes [2], but it is currently unclear how this translates to differences in mitochondrial structure. In agreement with a previous study, we observed significant morphological alterations in UC tissue along with reduced mitochondrial numbers, with possible further stratification by inflammation state [3]. These abnormalities point to severe mitochondrial dysfunction and contribute to our understanding of UC as a possible mitochondriopathy. As mitochondria are the site of oxygen-consuming processes such as oxidative phosphorylation [9], reductions in mitochondrial number might be postulated to increase oxygen levels in the gut, leading to alterations in the intestinal microbiota to favor facultative anaerobes and the development of dysbiosis [34, 35].

Our data points to dysregulated NAD^+^ metabolism and altered mitochondrial morphology as possible features of UC. However, they do not reveal a causal relationship between these features in the context of UC i.e. whether reduced NAD^+^/NAM ratio drives alterations in mitochondrial morphology, vice versa or both. Reductions in NAD^+^ levels have been associated with multiple contexts such as aging, neurodegeneration and metabolic disease [36]. Importantly, enhancement of NAD^+^ levels through provision of NAD^+^ precursors and targeting of specific enzymes involved in NAD^+^ biosynthesis/degradation has been shown to reverse several of these pathologies including mitochondrial dysfunction, pointing to a causal role of NAD^+^ metabolism in regulating these processes [15, 37].

One of the key ways by which NAD^+^ levels regulate mitochondrial metabolism is its effect on sirtuins, a family NAD^+^-consuming protein deacylases [36]. Reductions in NAD^+^ levels lead to reduced activities of SIRT1 and SIRT3, resulting in reduced mitochondrial biogenesis and altered mitochondrial morphology (for SIRT1) as well as mitochondrial protein hyperacetylation (for SIRT3) [38–41]. Patients with PARP1 hyperactivation also show reduced levels of mitophagy due to defective SIRT1 activity [42]. It is thus possible that reductions in NAD^+^ levels lead to alterations in mitochondrial morphology in a sirtuin-dependent manner.

However, the converse (mitochondrial dysfunction driving NAD^+^ depletion) can also occur. The reduced form of NAD^+^—NADH—serves as an important carrier of metabolic energy to complex I of the mitochondrial electron transport chain (ETC), where it is oxidized back to NAD^+^ [15]. Deletion of complex I has been reported to reduce the NAD^+^/NADH ratio, subsequently impairing SIRT3 activity [39]. Therefore, the overall picture is likely complex, whereby mitochondrial state and NAD^+^ metabolism are closely intertwined and can mutually affect each other. A mechanistic understanding of this relationship in suitable *in vitro* or *in vivo* models would thus be important in understanding the contribution of these features to UC.

In IBD, the role of NAD^+^ metabolism in disease pathogenesis is unclear [15]. NAMPT, the rate limiting enzyme of the NAD salvage pathway, is upregulated in IBD [43, 44]. Blockade of NAMPT ameliorated experimental colitis, suggesting this pathway as a potential therapeutic target [24, 25]. Interestingly, treatment with the NAMPT inhibitor FK866 did not affect levels of ATP but rather the activities of NAD^+^-catabolizing enzymes such as PARP1, SIRT6 and CD38 [24]. As mentioned above, CD38 activity is potentially pathogenic in IBD [30]. PARP1 and PARP2 have also been documented to promote colitis in mice [45, 46]. However, the roles of the SIRTs are not straightforward and sometimes contradictory. While multiple sirtuins (SIRT1,2,3,5,6) have been reported to exhibit protective effects on IBD [47–52], other sirtuins (SIRT1) can be pathogenic [53]. Together, the findings from our study and others support the need for further investigation of NAD^+^ metabolism in IBD.

Our study raises several interesting avenues for further investigation. Although our sample size was sufficient to pick out certain interesting metabolic phenotypes, further studies in a larger cohort will be needed to ascertain if these findings can encompass the heterogeneity of human UC [18]. It will also be interesting to investigate the relevance of NAD^+^ depletion to Crohn’s disease. We also note that these patients received various treatments, thus further studies focused on treatment-naïve patients will be helpful in discovering additional relevant metabolic pathways, particularly in the context of early disease. Further characterization of other metabolites in NAD^+^ metabolic pathways (e.g. ADPr derivatives, intermediates of NAD^+^ biosynthesis) will be critical in understanding the extent by which NAD^+^ metabolism is dysregulated in UC. Finally, it will be interesting to identify potential regulators of mitochondrial function in IBD, such as PGC-1α which can be regulated by NAD^+^ availability, and to understand how these regulators might also modulate intestinal inflammation [54].

In conclusion, we show that the colonic environment in human UC is marked by dysregulated metabolism and mitochondrial morphology. The altered metabolism is specific to UC patients displaying active inflammation and can be distinguished by a reduced NAD^+^/NAM ratio. We further show that UC tissues display altered mitochondrial morphology. Together, these suggest that NAD^+^ metabolism might be an attractive therapeutic pathway for targeting colonic inflammation.

## Supporting information

S1 Fig Additional metadata and further initial metabolomic analyses **(A)** Table showing treatment demographics of control and UC patients. **(B)** Pearson correlation analysis of the sum of all metabolite intensities for each mode vs initial weight of biopsy in all samples including outlier. **(C)** PCA analysis of all samples with outlier included. **(D)** Scree plot of PCA analysis as in Fig 1B–1D. **(E)** PCA analysis as in Fig 1C and 1D, but stratified by activity. Inactive patients included all controls as well as UC patients in remission. ^**†**^ indicates patient was taking sulfasalazine (not a 5-ASA but often considered one) for arthritis.
(TIF)

S2 Fig Graphs of other metabolites differentially regulated as in Fig 1E but not shown in Fig 2C–2E. Graphs depict mean ± SEM. *p<0.05, **p<0.01, ***p<0.001, ****p<0.0001 analyzed by Mann-Whitney test.
(TIF)

S3 Fig NAD, NAM and ADPr from Fig 2C–2E, with further separation of uninflamed samples into controls and UC uninflamed. Normalized abundance of **(A)** NAD^+^ and its degradation products **(B)** NAM and **(C)** ADPr indicated. Graphs depict mean ± SEM. *p<0.05, **p<0.01 by Kruskal-Wallis Test with Dunn’s correction for multiple comparisons.
(TIF)

S4 Fig PCA Analyses focusing on PC3 and PC4. (**A-D**) Principal component analysis (PC3 vs PC4) of metabolomic data comparing **(A)** Control vs UC biopsies, **(B)** uninflamed (control + UC uninflamed) vs inflamed biopsies, **(C)** Active vs Inactive (control + UC in remission) or **(D)** all samples stratified by age. (**E**) Loadings plot of PC3 and PC4 showing metabolites in top/bottom 5% of the loadings range for each PC. (**F**) Spearman correlation coefficients of the different clinical parameters (excluding outlier) performed using *Hmisc* R package and visualized the *corrplot* R package, with values having p>0.05 removed and ordered by hierarchical clustering. (**G**) Loadings plot as in (F), but for PC1 and PC2.
(TIF)

S5 Fig Quantitation of the EM micrographs as in Fig 4, but with samples stratified by inflammation. Uninflamed represent control + UC uninflamed. Graphs show **(A)** changes in the percent area occupied by the mitochondria between groups and **(B)** the number of mitochondria per 100 μm^2^ area, with no significant changes in **(C)** single mitochondrial area or **(D)** the roundness of mitochondria. Graphs depict mean ± SEM, *p<0.05 by Mann-Whitney test. Each point denotes the average of technical replicates for one control/patient.
(TIF)

S1 Table Patient demographics for the EM study, showing age (mean ± S.D.), gender, BMI (mean ± S.D.), biopsy location, duration of disease (mean ± S.D.), disease activity, extent of disease (mean ± S.D.), and treatment demographics. **p<0.01 by Mann-Whitney test. † indicates patient was taking sulfasalazine (not a 5-ASA but often considered one) for arthritis.
(TIF)
