# Supplementary figures and images for: Metabolic analyses reveal dysregulated NAD+ metabolism and altered mitochondrial state in ulcerative colitis
Source: PLoS One. 2022 Aug 17;17(8):e0273080. doi: 10.1371/journal.pone.0273080 (PMC9385040; doi:10.1371/journal.pone.0273080)

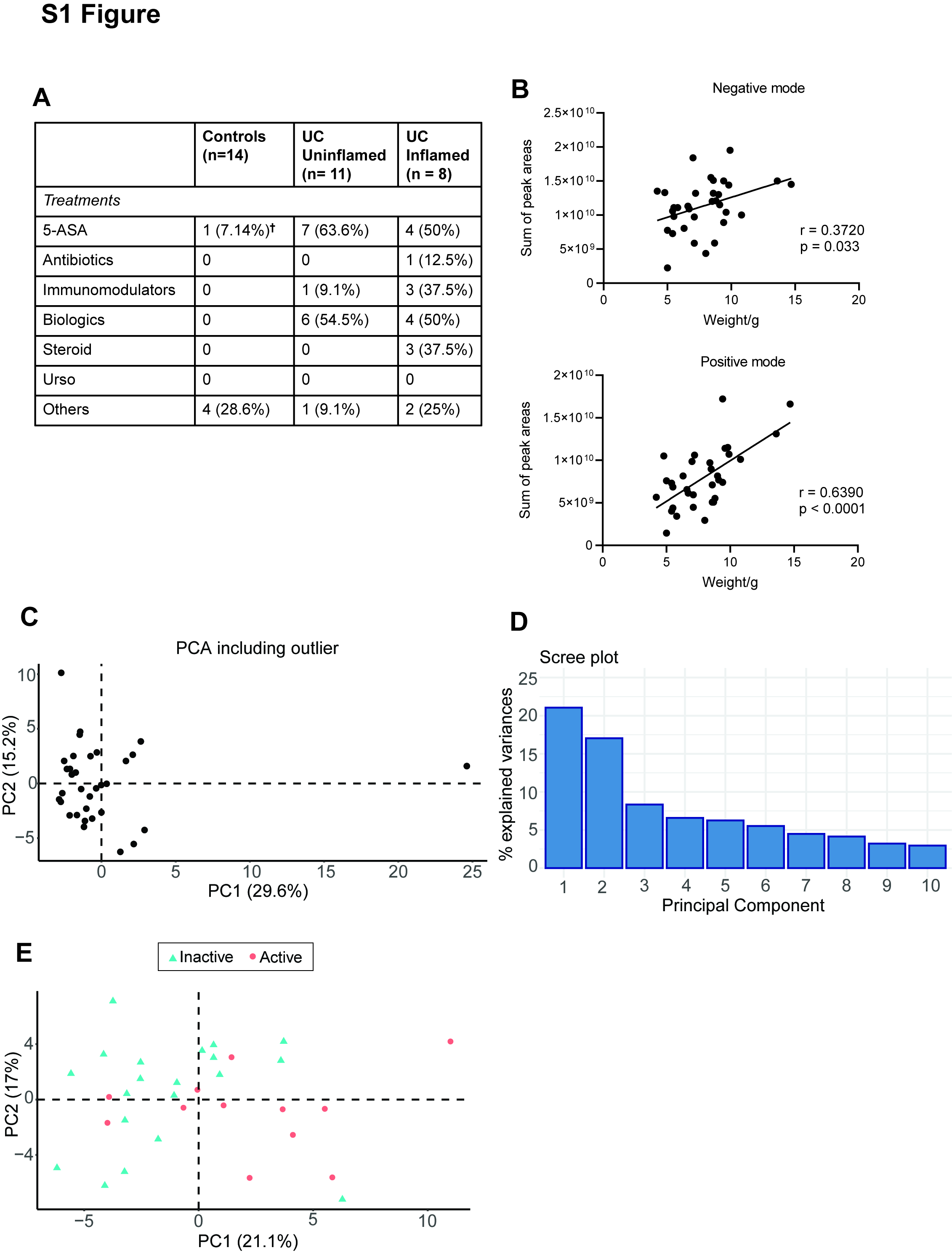

Supplement: S1 Fig — Additional metadata and further initial metabolomic analyses (A) Table showing treatment demographics of control and UC patients. (B) Pearson correlation analysis of the sum of all metabolite intensities for each mode vs initial weight of biopsy in all samples including outlier. (C) PCA analysis of all samples with outlier included. (D) Scree plot of PCA analysis as in Fig 1B–1D. (E) PCA analysis as in Fig 1C and 1D, but stratified by activity. Inactive patients included all controls as well as UC patients in remission. † indicates patient was taking sulfasalazine (not a 5-ASA but often considered one) for arthritis. (TIF) [file pone.0273080.s001.tif]

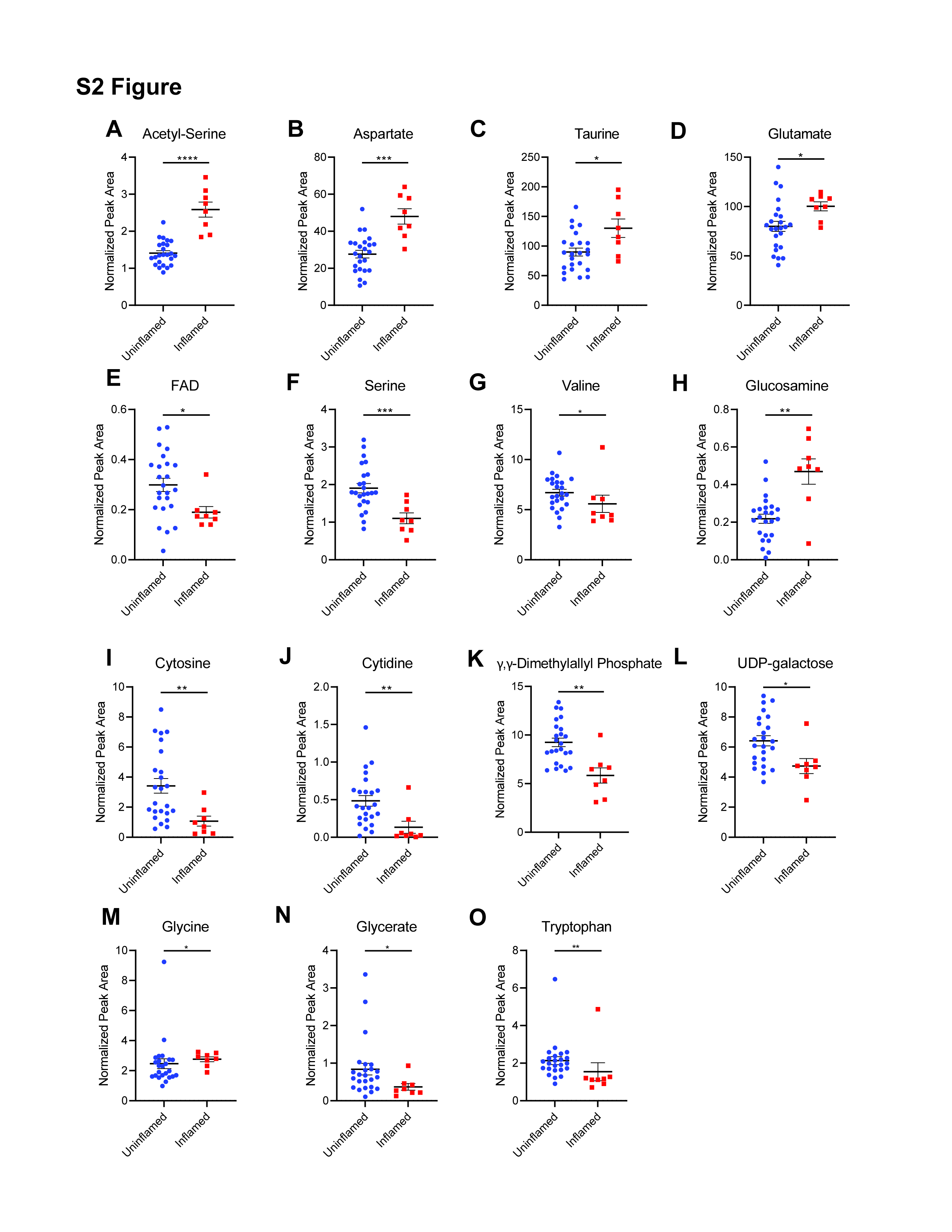

Supplement: S2 Fig — Graphs depict mean ± SEM. *p<0.05, **p<0.01, ***p<0.001, ****p<0.0001 analyzed by Mann-Whitney test. (TIF) [file pone.0273080.s002.tif]

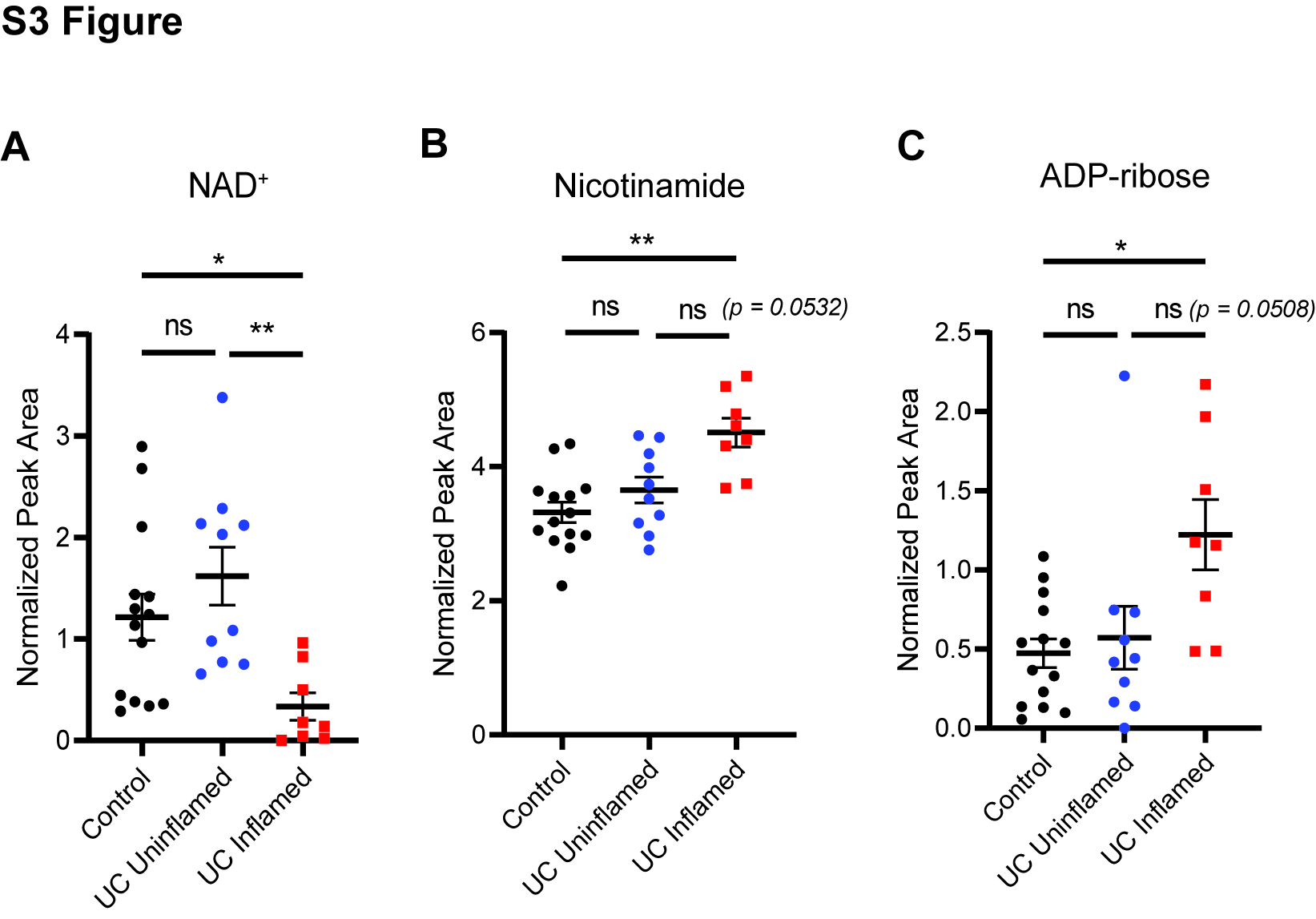

Supplement: S3 Fig — Normalized abundance of (A) NAD+ and its degradation products (B) NAM and (C) ADPr indicated. Graphs depict mean ± SEM. *p<0.05, **p<0.01 by Kruskal-Wallis Test with Dunn’s correction for multiple comparisons. (TIF) [file pone.0273080.s003.tif]

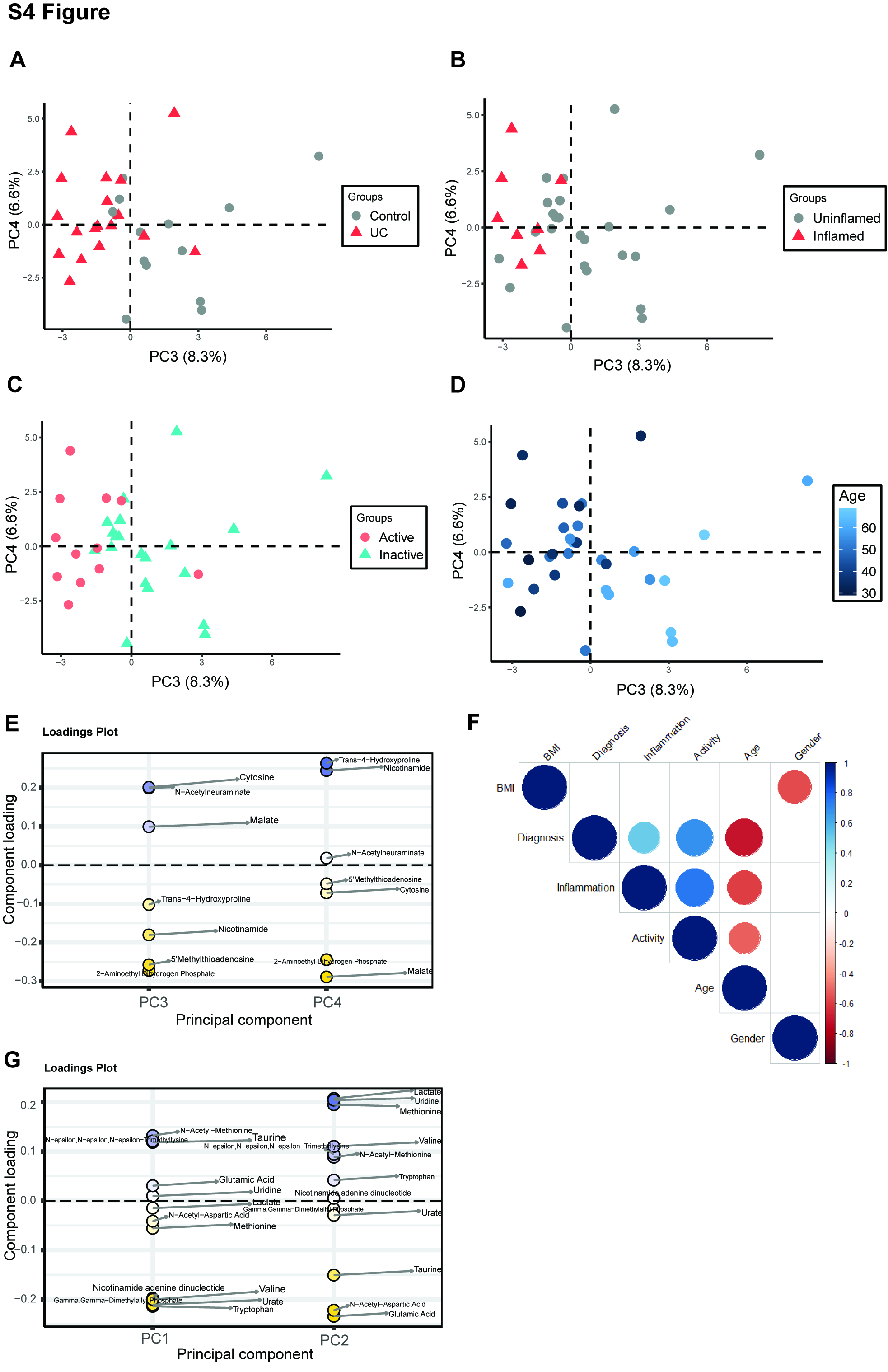

Supplement: S4 Fig — (A-D) Principal component analysis (PC3 vs PC4) of metabolomic data comparing (A) Control vs UC biopsies, (B) uninflamed (control + UC uninflamed) vs inflamed biopsies, (C) Active vs Inactive (control + UC in remission) or (D) all samples stratified by age. (E) Loadings plot of PC3 and PC4 showing metabolites in top/bottom 5% of the loadings range for each PC. (F) Spearman correlation coefficients of the different clinical parameters (excluding outlier) performed using Hmisc R package and visualized the corrplot R package, with values having p>0.05 removed and ordered by hierarchical clustering. (G) Loadings plot as in (F), but for PC1 and PC2. (TIF) [file pone.0273080.s004.tif]

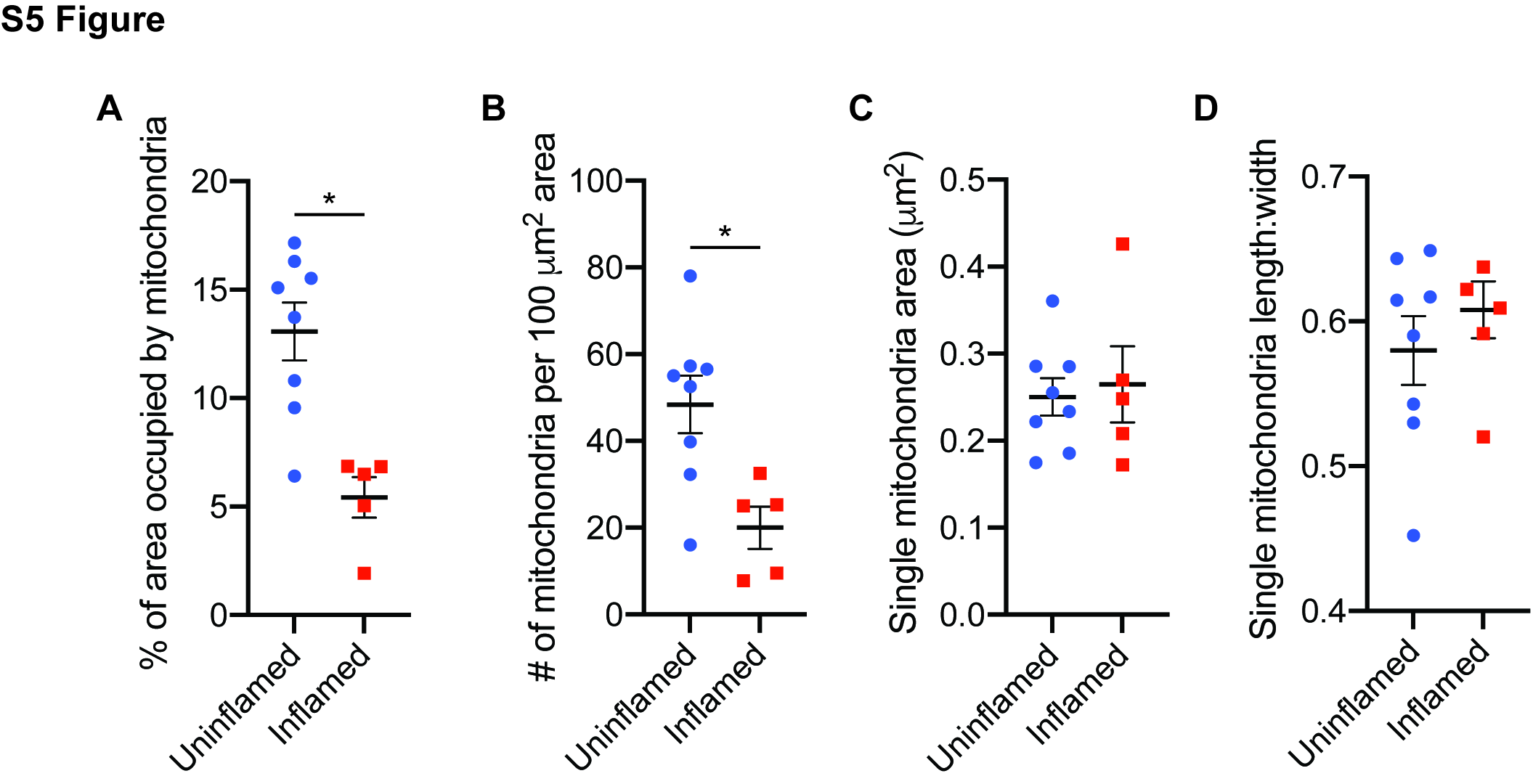

Supplement: S5 Fig — Uninflamed represent control + UC uninflamed. Graphs show (A) changes in the percent area occupied by the mitochondria between groups and (B) the number of mitochondria per 100 μm2 area, with no significant changes in (C) single mitochondrial area or (D) the roundness of mitochondria. Graphs depict mean ± SEM, *p<0.05 by Mann-Whitney test. Each point denotes the average of technical replicates for one control/patient. (TIF) [file pone.0273080.s005.tif]

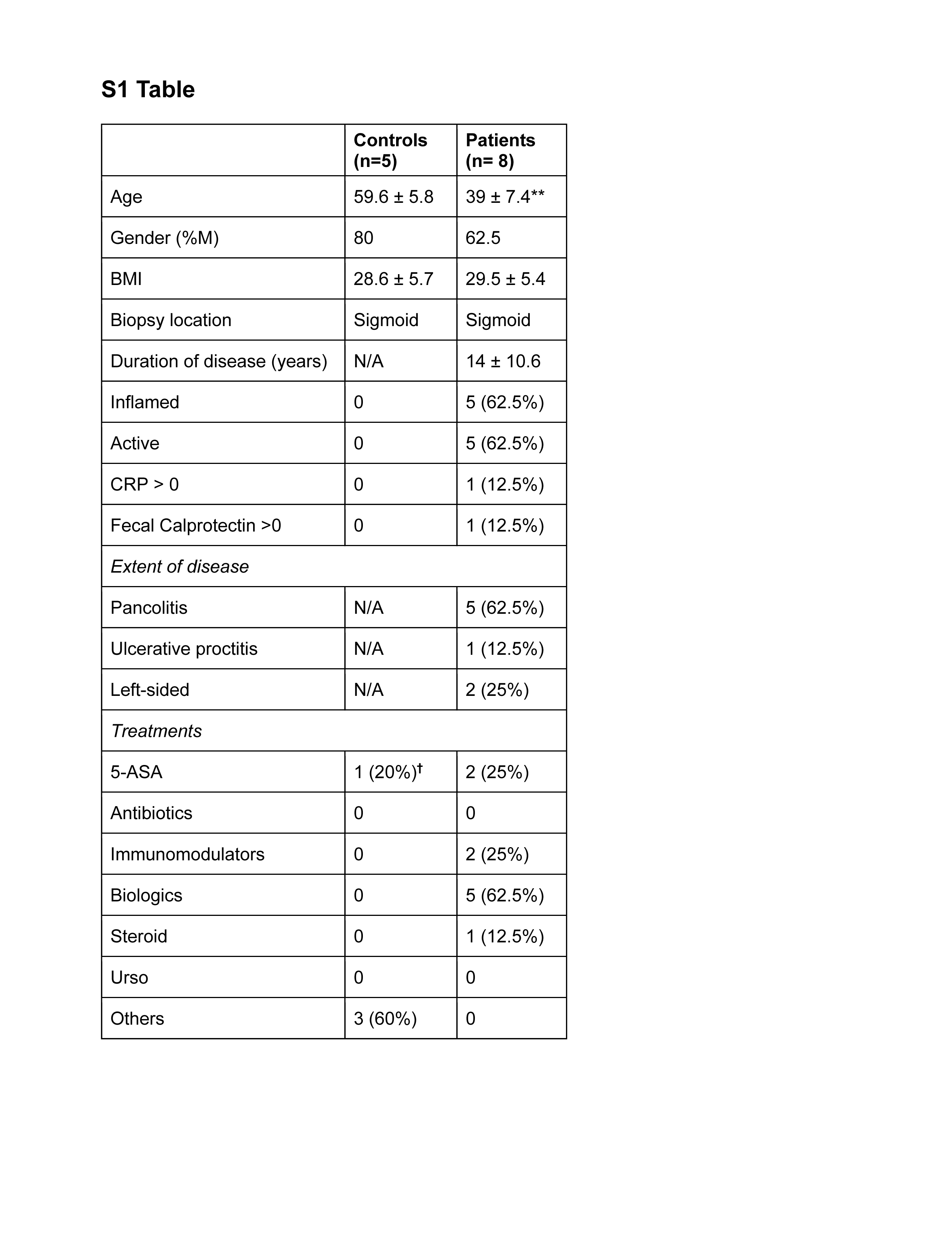

Supplement: S1 Table — **p<0.01 by Mann-Whitney test. † indicates patient was taking sulfasalazine (not a 5-ASA but often considered one) for arthritis. (TIF) [file pone.0273080.s006.tif]
